# Immunotherapy of HPV-associated cancer: DNA/plant-derived vaccines and new orthotopic mouse models

**DOI:** 10.1007/s00262-015-1734-0

**Published:** 2015-07-03

**Authors:** Aldo Venuti, Gianfranca Curzio, Luciano Mariani, Francesca Paolini

**Affiliations:** HPV-UNIT, Laboratory of Virology, Regina Elena National Cancer Institute, Via E. Chianesi 53, 00144 Rome, Italy

**Keywords:** PIVAC 14, HPV, Plant vaccine, DNA vaccine, Cervical cancer, Head/neck cancer

## Abstract

Under the optimistic assumption of high-prophylactic HPV vaccine coverage, a significant reduction of cancer incidence can only be expected after decades. Thus, immune therapeutic strategies are needed for persistently infected individuals who do not benefit from the prophylactic vaccines. However, the therapeutic strategies inducing immunity to the E6 and/or E7 oncoprotein of HPV16 are more effective for curing HPV-expressing tumours in animal models than for treating human cancers. New strategies/technologies have been developed to improve these therapeutic vaccines. Our studies focussed on preparing therapeutic vaccines with low-cost technologies by DNA preparation fused to either plant-virus or plant-toxin genes, such as saporin, and by plant-produced antigens. In particular, plant-derived antigens possess an intrinsic adjuvant activity that makes these preparations especially attractive for future development. Additionally, discrepancy in vaccine effectiveness between animals and humans may be due to non-orthotopic localization of animal models. Orthotopic transplantation leads to tumours giving a more accurate representation of the parent tumour. Since HPV can cause cancer in two main localizations, anogenital and oropharynx area, we developed two orthotopic tumour mouse models in these two sites. Both models are bioluminescent in order to follow up the tumour growth by imaging and are induced by cell injection without the need to intervene surgically. These models were utilized for immunotherapies with genetic or plant-derived therapeutic vaccines. In particular, the head/neck orthotopic model appears to be very promising for studies combining chemo-radio-immune therapy that seems to be very effective in patients.

## Introduction

All cervical cancers and precancerous lesions (CIN III) are caused by HPV infection. Since the early findings in the 1970s of HPV in cervical cancer by zur Hausen’s group, HPV has been linked to other anogenital cancers including vulvar, penile, and anal neoplasia. Recently, high-risk HPV (HR-HPV) infection has been detected in head and neck cancers (HNCs), with high frequency in oropharynx and tonsils, unveiling another aetiological factor in addition to tobacco and alcohol [1].

Screenings for pre-malignant lesions of the cervix and more recently HPV tests have led to a decrease in cervical cancer in developed countries with high costs for follow-up visits. Unfortunately, vaccination and screening programs for cervical cancer in developing countries are very difficult, and for this reason, cervical cancer represents a major cause of death in women. Furthermore, there is yet no valid screening to identify early HPV-positive (or negative) HNCs. HPV-negative HNCs are declining, which may be related to less tobacco consumption, whereas HPV-positive oropharyngeal cancer is increasing with an expected higher rate than cervical cancer in the 2020s in the USA [2].

Although the natural history of HPV-associated oral cancer is still not well known, similarities exist with cervical cancer, such as the number of cancers increases with increasing number of sexual partners, and therefore, some paradigms of HPV function can be adapted for HNC. From this perspective, plausibly preventive vaccines against HPV infection will also work in preventing oropharyngeal cancers. A population-based study in Costa Rica showed that HPV prevalence in the oropharynx of vaccinated women four years after follow-up was much lower among the vaccine arm compared with the control arm, suggesting that the vaccine strongly protects against oral HPV infection, and therefore may prevent also HPV-associated oropharyngeal cancers [3].

Currently available preventive vaccines against HPV are based on virus-like particles (VLPs) prepared by recombinant expression and assembly of the major capsid protein L1. Merck’s yeast-made Gardasil and GSK’s insect-cell-made Cervarix are VLP-based vaccines both highly effective/powerful but not cost-effective; consequently, their use is hampered in developing countries where HPV accounts for ~85 % of both annual cervical cancer cases (~500,000 cases worldwide) and deaths from cervical cancer (~300,000 worldwide) [4]. The costs and cost-effectiveness of these vaccines will remain a problem even if the second-generation vaccines will include wider coverage like combination of more types of HPV L1 vaccines (Gardasil 9-valent), or chimeric L1/fragment L2 vaccines, or L2-based vaccines [5]. Neither of these vaccines, Gardasil or Cervarix, have any effect on established infections; thus, million people already infected need a therapeutic intervention. No specific/effective pharmacological treatments exist; thus, immunotherapy can be a valid option, particularly because the HPV lesion/cancer possesses strong tumour-associated antigens (TAA) represented by the major oncoproteins E6/E7 always expressed and necessary to maintain the transformation status. Together with a broad spectrum of preventive HPV vaccinations, also treatments of persistent infections, precancerous lesions, and early-stage tumour by immunotherapy could lessen the burden of HPV disease.

Vaccine-mediated immune strategies can be directed towards at least two different steps of the HPV oncogenic process: primary infections by prophylactic vaccines and established infections by immunotherapy targeted to early proteins, in particular E6–E7 oncoproteins. Treatments by immunotherapy could have an immediate impact on HPV-associated cancer, whereas prophylactic vaccines will take decades to lead to death reduction due to this pathology. Therapeutic vaccines are aimed to eradicate or to reduce already infected cells. Indeed, E6 and E7 proteins are always associated with tumour cells and are not expressed on the cell surface, making an antibody-based immune response unlikely to eliminate already infected cells. In HPV-infected/transformed keratinocytes, the constitutive expression of E6 and E7 results in their peptides being presented on the surface of cells by MHC class I molecules. These infected cells can be now recognized by cytotoxic T-lymphocytes (CTL) that play a pivotal role in clearance of virus-infected cells.

The primary CTL response is activated by the presentation of foreign antigen on the surface of antigen-presenting cells (APC) within MHC class I molecules. Since HPV does not infect primarily APC to allow usual class I processing, HPV antigens at some stage of infection or following cell trauma are released from infected cells and APCs are able to process these exogenous protein to produce class I-linked peptides and prime CTLs. In addition, APCs present HPV antigens also within class II MHC molecule priming in particular CD4+ helper T cells. CD4+ T cells are essential for both a valid cellular and humoral immune response. The conditions of T-helper lymphocyte activation decide their phenotype resulting in two populations, Th1 and Th2. The equilibrium between Th1 and Th2 is often altered in HPV infection with reduced Th1 and increased Th2 response associated with lesion progression [6]. On the other hand, induction of specific CTLs and T-helper has been recognized in patients regressing from natural HPV infection [7]. Thus, similar immunological responses must be elicited by a therapeutic vaccine in order to eliminate HPV-infected/transformed cells.

Therefore, the basic principle of therapeutic vaccine is to stimulate cytotoxic T cells against the target infected cells together with an up-regulation of MHC Class I expression. Since the first human trials on late-stage cervical cancer, it was clear that by reason of the immunosuppressive environment generated by tumours in cases of advanced disease, an immunological anti-tumour effect could be effective only together with ordinary therapy. This immunosuppressive milieu (partially related to HPV action) involves: (1) the disruption of antigen processing and presentation machinery by altering the MHC class I and TAP 1–2 expression [8]; (2) the recruitment of immunosuppressive T regulatory cells (Treg) to dampen effector T cell activity; and (3) the chemokine production altering T cell homoeostasis and increasing the sensitivity of effector T cells to apoptosis [9]. Thus, a valid therapy in late stage of disease should not only induce cytotoxic activity, but also break the immunotolerance/immunoescape of the tumour. To achieve this result, we can benefit from the existence of known precursors to the tumour of cervix, anus, or vulva in which this immunosuppressive environment is less active. In alternative, we can modify the vaccine/immunotherapy schedule in order to attract effector cells to the site of action. Experimental vaccines developed against E6–E7 oncogenes have shown some effectiveness in animal models and human trials. However, given that most of the cervical disease burden as well as cancer occurs in developing countries, the problem of costs and smaller size of the market will be once again an issue that should be settled by any licensed vaccines [10, 11]. In an attempt to produce a valid immunotherapy possibly at low cost against HPV-associated lesion/cancer, our studies have focused on the development of low-cost platforms, such as DNA and plant biotechnologies. To produce tailor-made and potentiated formulations, plants will be utilized as bio-factories or “mines” of immune stimulators. More importantly, and regardless of the formulation type, clinical trials require pre-clinical assays and models to predict the treatment efficacy. In particular, pre-clinical models resembling human pathologies are necessary. Our studies were therefore focussed on developing orthotopic models of HPV lesions/cancers.

## DNA vaccines

DNA-based vaccines are increasingly considered to be a promising therapeutic approach against malignancies as they are safe, easy to produce at high purity, and provide stable expression of the encoded antigen [12]. This type of vaccine-based approach may also have adjuvant functions, such as plasmid DNA harbouring unmethylated CpG motifs, which can be recognized by Toll-like receptors (TLR)-9 stimulating innate immunity [13, 14]. DNA vaccination is producing non-live, non-replicating, non-spreading antigens capable of inducing both CTL and Th immunity, as well as B cell immunity. In addition, multiple DNA administrations are feasible without triggering an immune response against the DNA plasmid. This method may therefore be particularly useful in the context of therapeutic cancer vaccination, where repeated vaccinations are often required to boost effective T cell responses. Several pre-clinical and clinical DNA vaccine studies against HPV-induced malignancies have been conducted, and clinical trials are described elsewhere [10, 11].

The association between antigens and proteins capable of inter- and intra-cellular transport has been shown to enhance the spread of antigens encoded by DNA vaccines. Our group has developed strategies aimed at enhancing antigen processing through the MHC class I or II pathways. Fusion genes consisting of HPV16 E7 oncogene fused to the genes for ubiquitin or the invariant chain (Ii) were produced, in order to increase the presentation of E7-derived peptides by MHC class I or II molecules, respectively. DNA vaccination of C57BL/6 mice was performed with these fusion genes, and the animals were subsequently challenged with E7-positive tumour cell lines expressing different levels of MHC class I molecules. A number of animals were protected from tumour challenging by the E7-Ii fusion gene [15].

A harmless version of the HPV16 E7 gene, the E7GGG mutant that was produced by introducing three amino acid substitutions at the binding site for pRb [16], was fused to the 3’ end of a capsid protein (CP) of a plant virus producing another DNA fusion vaccine [17]. Indeed, CPs, in particular those of the potato virus X (PVX), have been largely used as carrier proteins for a range of epitopes of animal pathogens [18]. Moreover, as they act as primary antigens in humans with the ability to aggregate in structures activating CD4+ T responses [19], CPs assume the role of pathogen-derived dominant antigens that could enhance the immunogenicity of poor/silent determinants [20]. The efficacy of different DNA constructs was assayed in a mouse model, the TC-1 cell line. TC-1 cells are derived from immortalized mouse cells expressing HPV16 E6/E7 and Ras oncogenes and are able to produce tumours in syngeneic C57BL6 mice [21]. This model is widely used to assess the efficacy of therapeutic anti-HPV vaccines. In therapeutic vaccination experiments with pcDNA/E7GGG-CP fusion constructs, a greater number of mice challenged with tumour-inducing E7-expressing TC-1 cells are protected (60 %) compared with pcDNA/E7GGG alone. This difference indicates that engineering the intracellular pathway for antigen presentation produces a valid therapeutic response, at least in mouse models [17].

HPV collaborates with molecules implicated in the immune surveillance process, depleting apoptosis and creating an immunosuppressive tumour environment associated with an ineffective immune response to the viral early antigens, E7 and E6 [11, 22]. Consequently, approaches were developed to boost the “strength” of vaccines against the targeted tumour antigens in order to utilize new genes/proteins with immunomodulatory/stimulatory properties. An example of these vaccines fused to immunostimulant proteins that has reached phase I clinical trial in CIN 2/3 patients is the DNA vaccine encoding a signal sequence for the endoplasmic reticulum linked to an attenuated form of HPV16 E7 gene fused to Hsp70 (Sig/E7detox/Hsp70). Results of this clinical trial demonstrated safety and tolerability of the vaccine with histological regression in 3 out of 9 patients treated with the highest dose. Although this histological regression was not statistically significant, some HPV-specific T cell responses elicited in patients offer promising therapeutic perspective [23]. Indeed, same vaccine boosted with the recombinant vaccinia virus encoding HPV16/18 E6/E7 fusion protein TA-HPV with or without imiquimod is now utilized in another clinical trial started in 2008 with expected results in 2016 [24]. Possible autoimmune responses induced by Hsp70 are one of constraints in using this kind of vaccines. However, human HSP70 instead of mycobacterium tuberculosis HSP70 significantly decreases autoimmune disease risk [25]. Nevertheless, improvement in therapeutic HPV vaccines in clinical trials has to take into account any possible problem that may be amplified by combination strategies of different therapeutic modalities [26].

In seeking novel immunostimulatory sequences with less limitations to their clinical use (i.e. autoimmune disease), our group has created a new strategy using plant-derived carriers, represented by “Ribosome inactivating proteins” (RIPs). RIPs are an inhibitor family of cellular protein synthesis, and they are found in different organs of many plant species where they have regulatory and defensive roles [27]. They are N-glycosidases that remove specific adenine bases in 23S/25S/28S rRNA triggering protein synthesis arrest at the translocation step and inducing cell death. Due to its toxic activity, RIPs together with targeting molecules (i.e. monoclonal antibodies) have shown great potential for their use as selective killing agents of tumour, immune or nerve cells in medicine. Unfortunately, these chimerical toxins did not turn out to be perfect clinical tools as their clinical application was hampered by immunogenicity of the toxin moiety and by non-specific toxicity leading to vascular leak syndrome [28]. Regardless of cytotoxicity by protein synthesis arrest, other RIPs biological characteristics could contribute to the design of therapeutic anti-tumour vaccines. In particular, RIPs are characterized by: (1) a high antigenicity that could be helpful in boosting immune response against fused tumour/viral antigens; (2) the ability to modulate non-specific and innate immune functions affecting NK, CD4+, and CD8+ T cells, and cytokine production; and (3) the induction of inflammation and apoptosis [29]. Saporins belong to a multigene family of single chain (type 1) RIPs, which are formed in various organs of Saponaria officinalis (soapwort). Saporins have been used to develop immunotoxins against cancer cells for their catalytic activity once penetrated in the cytoplasm. They are stable proteins resistant to derivation and conjugation processes. In addition, independently of inhibiting translation, saporin has been shown to induce cell death via apoptosis in a variety of cellular models [29]. A fusion construct of a non-toxic saporin mutant (SAP-KQ) with HPV16 E7GGG gene was generated. This construct affected tumour growth of E7-expressing TC-1 cells inducing E7-specific immunoglobulins (IgG), CTLs, and delayed-type hypersensitivity (DTH) [30]. In the context of DNA-based vaccination, these data demonstrate that mutant plant genes are potentially capable of improving the poor immunogenicity of tumour-associated cancer antigens fostering the development of new cancer immunotherapy. In addition, our E7 vaccines were utilized in prime/boost schedule with recombinant fowl-pox virus showing some promising results in mice [31].

A further improvement in fusion DNA vaccines could be achieved by modifying administration routes as well as schedules of vaccination. The introduction of electroporation (EP) represents a major advancement in DNA vaccination. EP consists of applying short electric pulses to the site of vaccination after intramuscular or intradermal plasmid DNA administration. EP increases plasmid uptake and generates a local inflammatory cell infiltrate, leading to a stronger immune response to the vaccine. In regards to the comparison of EP safety after DNA vaccination with that of DNA delivered without EP, there is no increased risk of toxicity nor integration of the plasmid DNA into the genome of the host cell. Indeed, a recent Phase I clinical trial conducted by EP of E6/E7 HPV16 and 18 DNA vaccine ascertained its completely safe administration in humans [32]. For this reason, our E7-SAP vaccine will be used via EP in pre-clinical models.

Literature data, along with our results on different HPV DNA vaccines, support the use of DNA vaccines as valuable tools in therapeutic development of HPV vaccine. EP protocols or heterologous DNA prime and viral vector-based boosts could increase immunogenicity of HPV TAA. In addition, DNA vaccine efficiency in clinical setting will be further enhanced by combination with Treg depletion, which may be a valid tool in avoiding immune escape [33, 34].

## Plant-derived vaccines

Originally, vaccines from plants were developed with the idea of edible vaccines [35, 36]. However, it became soon clear that this concept was not feasible because the administration of vaccines to humans requires standardizing doses, and establishing measures of quality control for purification and formulation [36]. The production of plant-based vaccines against different pathogens has been developed over the last few decades in prophylactic and therapeutic settings and is reviewed elsewhere [37]. Recently, the first Food and Drug Administration qualified clinical study of a plant-derived vaccine on non-Hodgkin’s lymphoma has demonstrated that plant proteins are safe just like biologicals from other sources, when given as parenteral administration. Additionally, immune responses to the specific antigenic determinants were observed in 66 % of patients, and not towards xenogeneic plant antigens [38]. Therefore, plant-produced vaccines can be utilized in humans following standardized procedures. In this field, the production of plant-based vaccines against HPV was developed for producing VLP for prophylactic vaccines as well as E6/E7 antigens for immunotherapy. The major accomplishments in plant-produced VLP prove that HPV capsid protein L1 can self-assemble in transgenic plants [39, 40], and the derived VLP induces immune responses in rabbit models [41]. Initially, low yields of plant-produced VLPs were observed; however, recent attempts via agro-infiltration-mediated transient expression or via transplastomic (chloroplast) expression showed higher yields of HPV-16 L1 and VLPs [42].

Our research group was the first to investigate plant-derived therapeutic vaccines based on the HPV-16 E7 oncoprotein. A recombinant PVX vector successfully induced transient expression of the HPV16 E7 protein in Nicotiana benthamiana plants. Specific immune responses were induced in mice by tobacco plant extracts containing E7 protein. These immunological responses induced protection from tumour growth of E7-expressing C3 cell line coupled to strong cytotoxic T cell responses [43]. The immunological activity of plant-produced vaccine was hampered by the low level of E7 in the tobacco extracts but, by forcing E7 to the secretory pathway, it was possible to obtain an enhanced expression of the protein. This higher level of E7 within the tobacco extracts increased immunological responses and therapeutic vaccine effectiveness in the same mouse model [44]. It was clear from these experiments that, in the presence of plant extract, E7 antigens are effective in eliciting CTL response in the absence of any adjuvant molecules.

The word adjuvant comes from “adjuvare”, the Latin term for to help. Traditionally, when developing adjuvants, the main aim was to boost humoral immune responses, and as a result, most of the commonly used adjuvants are effective in raising serum antibody titres, without significantly eliciting Th1 responses or CTLs. Thus, a therapeutic setting in which strong CTL responses are elicited by adjuvant activity of plant extracts is particularly useful. Moreover, other studies showed plant extract immunomodulatory activity in vitro on dendritic cells [45].

To address potential concerns on using oncoproteins in vaccines, our group engineered mutations that eliminated any interactions with cell cycle governing proteins, the already mentioned E7GGG mutant, in order to attain a safe version of the oncoprotein. With the aim of further enhancing the therapeutic efficacy of E7GGG antigen, we constructed a new vaccine by fusing E7 antigen to a lichenase (LicKM), the beta-1,3-1,4-glucanase of Clostridium thermocellum and by producing it in tobacco plant using an innovative transient expression system, the pBID4 launch vector, which possesses the advantageous features of agrobacterial binary plasmids and those of plant viral vectors [46]. This plant-produced fusion E7GGG/LicKM antigen was evaluated as a possible therapeutic vaccine against HPV-induced tumours in TC-1 mouse model [47]. Data from induction of E7-specific IgG, DTH, and ELISPOT indicated that E7GGG/LicKM fusion proteins were highly immunogenic, capable of inducing both humoral and cell-mediated immune responses, superior to those generated by immunization with E. coli-produced E7GGG. In addition, during the administration of vaccine preparations without the use of adjuvants, our group observed that only tobacco-produced fusion proteins were able to prevent tumour growth. This observation indicates that LicKM fusion proteins play a key role in activating both innate and adaptive antigen-specific immune responses.

The therapeutic capabilities of these fusion antigens were further explored by starting vaccinations when TC-1 tumours were already palpable, and by assessing overall survival. The outcome of this study clearly demonstrated that this plant-produced LicKM-E7GGG fusion antigen is a valid therapeutic vaccine candidate by curing established experimental tumours in mice, as well as having a striking effect on the overall survival of treated animals [48]. Through metal-ion affinity chromatography and gel filtration, the production of this fusion protein with 99 % purity was scaled up to 100 mg/Kg biomass, which is an appropriate quantity for industrial production [49]. Thus, the possible use of this fusion vaccine in clinical trials is expected.

Our group investigated also an alternative production technique of E7GGG protein by recombinant chloroplast of Chlamydomonas reinhardtii, a well-characterized unicellular alga [50]. E7GGG was expressed to levels of 0.12 % of total soluble protein (TSP). Chlamydomonas reinhardtii E7 extracts or purified protein plus QuilA adjuvant was injected in C57BL/6 mice to produce immunological responses. These preparations induced specific anti-E7 IgGs and E7-specific T cell proliferation in mice as well as tumour protection after challenging with E7 expressing TC-1 tumour cell line. This was the first successful expression of a soluble E7 in plants. Moreover, the efficacy of the alga extracts in the absence of any adjuvant molecules demonstrated that even algae possess an immune-enhancing activity as shown for tobacco plants.

Other data about adjuvant activity of plant components come from a study on the synthetic shuffled HPV-16 E7 (16E7SH) that has no transforming function [51]. This 16E7SH was fused to the Zera^®^ peptide, a self-assembly domain of the maize gamma-zein seed storage protein, which drives recombinant proteins into endoplasmic reticulum. This fusion protein was effective in mice vaccination but, more interestingly, even a mixture of Zera and 16E7SH was able to induce enhanced immune responses, suggesting an adjuvant activity for the Zera^®^ component unrelated to the construct. Thus, we can state that different plant components have common immune-potentiating effects on therapeutic vaccines, which further consolidate the use of plant-based platforms in preparing vaccines. Currently, there are ongoing studies investigating and characterizing the presence of molecules with adjuvant activity bound to plant-produced E7 antigen aiming to validate and explain the underlying immune-enhancer effects of these vaccines. These analyses will achieve new insights of adjuvant molecules interacting with E7.

Future studies will focus on producing a fusion protein E7GGG/SAP not only in tobacco plants, but also in root cultures that are emerging as valid production tools [37]. Given the effectiveness of the E7GGG/SAP DNA vaccine in therapeutic setting, we reason that further developments of this vaccine will be a heterologous DNA prime/matched-protein boost scheduling, in order to increase the efficacy. Plant-based production of therapeutic vaccines against HPV diseases shows great potential for the future as these vaccines can be produced at high yield via transient expression and possesses anti-tumour and immune-enhancing activity at least in animal models. The possibility to scale up the production of antigens makes this technology suitable for effective translation into clinic.

## New orthotopic mouse models

Therapeutic programs for persistently infected individuals beyond the help of prophylactic vaccines are needed. The induction of specific immune responses to the non-structural oncoproteins of HPV can be achieved in humans and animals with appropriate immunotherapy. However, most of the developed therapeutic strategies are more effective for curing HPV transplantable tumours in animal models than for treating human HPV cancers. One of the possible causes of this discrepancy is represented by the non-orthotopic localization of the experimental animal models. Indeed, most of well-studied animal models are based on subcutaneous/intramuscular challenging of C57BL/6 mice with HPV16 expressing tumour cells, such as the embryonic C3 line or the epithelial TC-1 line. These models were useful in evaluating the safety of therapeutic vaccines and in examining whether they can induce HPV-specific immune responses that can eradicate a transplanted tumour. However, these models are quite different because E6 and E7 genes are under the control of a retroviral promoter in TC-1 cell line, while the natural promoter controls the expression of HPV genes in C3 cell line. Thus, this difference could be relevant in estimating the effectiveness of therapeutic vaccines.

It has been observed that an orthotopic transplantation, i.e. a tumour transplantation into the same body site from which the primitive tumour derived, represents more accurately the parental tumour. Since HPV can cause cancer in two main areas, anogenital and oropharynx area, and both sites appear susceptible to immunotherapy, we focussed our research on orthotopic mouse models of cancer in these two sites [52]. In order to follow up the tumour growth without surgical intervention, both models were engineered to be bioluminescent for imaging.

**Fig. 1:**
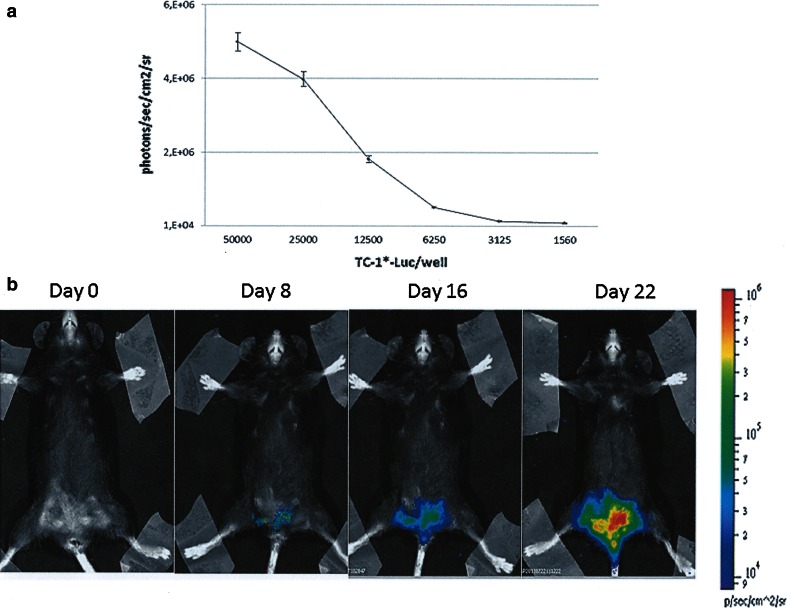
Genital mouse model of HPV-associated cancer. TC-1* cells were infected with Lenti-Luc vector to generate TC-1*-Luc cells. **a** Different concentrations of TC-1*-Luc cells (triplicates) were plated in 96-well plates, and D-luciferin (final concentration of 0.15 mg/ml) was added 15 min before monitoring. Bioluminescence was measured by imaging system and calculated as photons/s/cm^2^/sr. Each point is mean ± SD. **b** Diestrum synchronized eight-week-old female C57BL/6 mice were pre-treated with a spermicidal/detergent N9 (4 %), and after 6 h washed with phosphate-buffered saline before challenging with 5 × 10^4^ TC-1*-Luc cells. Genital tumour growth was monitored at the indicated time interval post-challenge. Bioluminescence was measured 15 min after intraperitoneal injection of D-luciferin by imaging system and quantified as photons/s/cm^2^/sr in a colour scale

The genital orthotopic model was originally developed by Decrausaz et al. [53], and we have reproduced the same model with our TC-1* star cells that are more aggressive than the parental TC-1 cells, giving 100 % of tumour upon inoculation in the flank of mice [48]. Briefly, TC-1* cells were infected by luciferase-encoding lentiviral vector producing bioluminescent cells for in vivo imaging of tumour growth (Fig. 1a). After in vitro passages, TC-1*-Luc cells were transplanted into the mouse vagina pre-treated with nonoxynol-9 (N9) in order to disrupt transiently the vaginal epithelium favouring the trapping of injected cells and the development of luminescent vaginal tumours. An example of this orthotopic HPV-associated mouse genital tumour is presented in Fig. 1b. This model was not further developed due to the arduous task of dealing with the experimental procedures, and in our hands, sometimes the tumour expands into the peritoneal area broadening the gap between the model and the human pathology. Moreover, TC-1 cells did not derive from the same anatomical area, and they did not represent a proper orthotopic transplantation. However, this model retains the immunological features of naturally occurring HPV tumours [53].

The second model of orthotopic HPV-associated cancer is based on oral localization. To the best of our knowledge, this is the first mouse model of HPV-associated HNC. Our orthotopic mouse model for HPV-related oral tumours derived from the murine AT-84 cell line, stemming from a spontaneous oral squamous cell carcinoma (OSCC) of C3H mice. This model represents more precisely the ancestral tumour probably due to the presence of the same microenvironment [54]. In particular, it resembles human cancer in histological features, frequent lung metastases, recurrences, and successful surgery/chemo/radiotherapy.

**Fig. 2:**
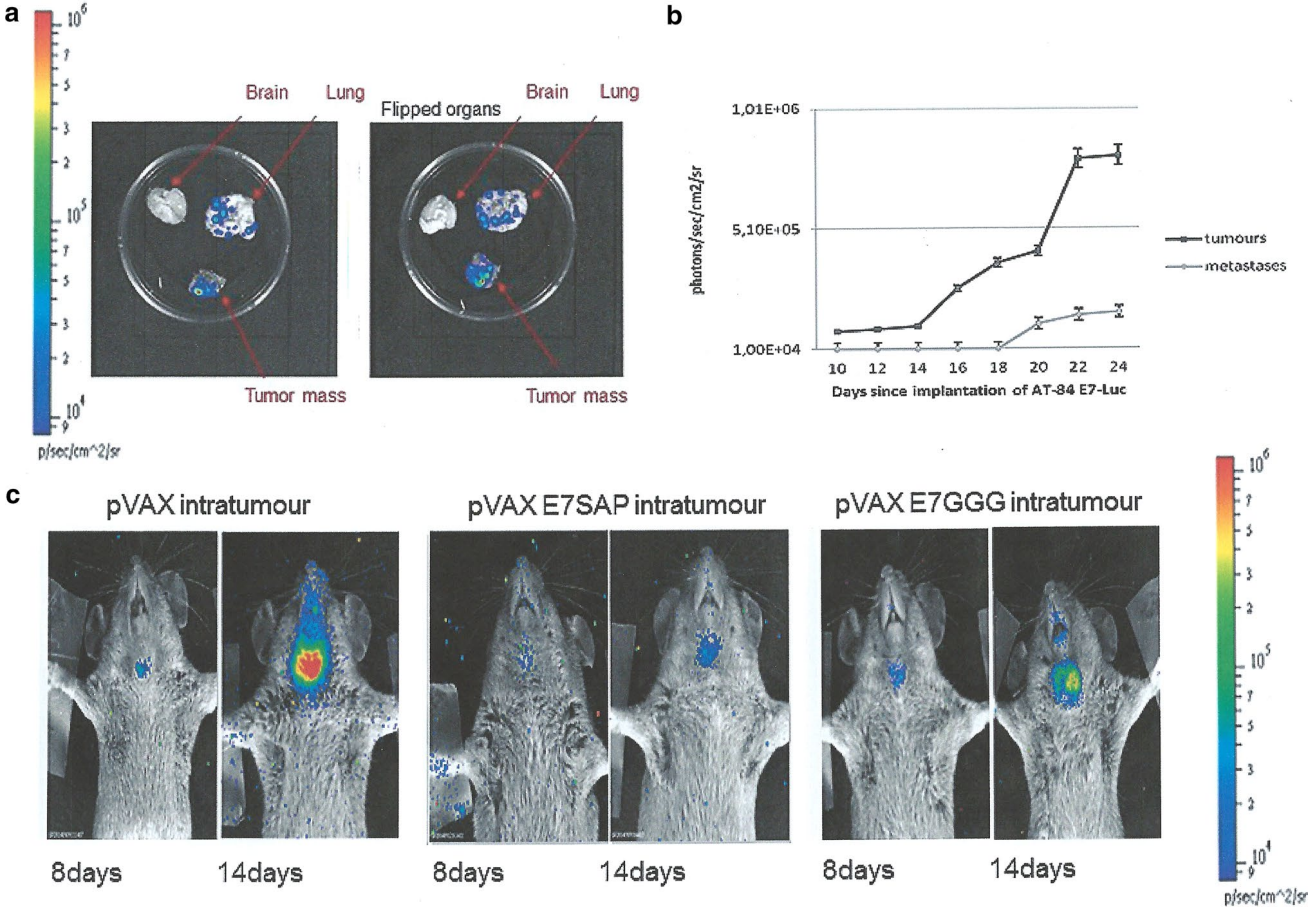
Head and neck mouse model of HPV-associated cancer: intratumoural therapy. **a** The presence of bioluminescent metastases in explanted organs of mice carrying the AT-84 E7 Luc tumours. Bioluminescence was measured by imaging system after addition of D-luciferin. **b** Time-course of luciferase expression in AT-84 E7 Luc tumour and metastasis. Bioluminescence was calculated as photons/sec/cm^2^/sr, and data are mean ± SD of 5 animals. **c** Representative intratumoural treatment. Treatment with indicated DNA vaccine preparations was performed by a prime dose (100 µg) i.m. 3 days after AT-84 E7 Luc injection in the mouth pavement followed 6 days later by DNA vaccine inoculation (100 µg) within the tumour. Tumour growth was monitored at the indicated time intervals post-challenge. Bioluminescence was measured 15 min after intraperitoneal injection of D-luciferin by imaging system and quantified as photons/s/cm^2^/sr in a colour scale. pVax, empty vector; pVax E7SAP, fusion between HPV16 E7GGG and mutated Saporin; pVax E7GGG, HPV16 E7GGG gene

When considering all these aspects, this animal model is a valid tool for testing novel therapies. The newly developed AT-84 E7 model maintains all the properties of the parental cells with addition of HPV16 E7 oncoprotein expression. Once implanted, the stable expression of this oncogene is maintained, not only within the tumour, but also in distant metastases (Fig. 2a, b) [55]. This result further supports the utility of this orthotopic mouse model for HPV-related human oral cancer, as E7 expression is always maintained in oral human cancer and metastases. Indeed, E7 protein expression levels are similar to those observed in naturally occurring HPV tumours. The low expression level of E7 in AT-84 E7 tumours compared with other non-orthotopic models (i.e. C3 and TC-1) may provide a more realistic picture of the natural condition of HPV-related tumours. Indeed, the great difference between the high expression levels of E7 in C3 and TC-1 models and the low levels in human cancers is a plausible explanation why several immunotherapies proven efficacious in C3 and TC-1 models failed to be effective in humans. Thus, AT-84 E7 cells may represent the best oral model to test the feasibility of therapeutic vaccination. This model was further improved by making AT-84 E7 bioluminescent by Lenti-Luc infection, allowing less invasive and more accurate follow-up [55].

Although this preliminary work regarding AT-84 E7 model for immunotherapy was not designed to assess immunological responses, the results provided information on the efficacy of heterologous prime-boost regimens compared with homologous prime-boost regimens, as reported in previous studies [23, 56]. Mice were vaccinated with different plant-derived formulations (DNA-based or plant-produced) to observe whether or not and to what extent anti-tumour activity was triggered in the AT-84 E7 model. These tailored formulations had already shown their ability to inhibit TC-1 tumour growth. Homologous or heterologous prime/boost regimens appeared to be less effective in AT-84 E7 than in TC-1 model, with strong effects on tumour burden and poor action on tumour onset [17]. In addition, AT-84 E7-Luc model not only permitted tumour mass measurement better than calliper, but also showed high capacity to detect cancer earlier. Indeed, a tumour mass was first evident on the eighth day post-transplantation when a palpable tumour is barely detectable.

However, several aspects need to be improved in order to have a completely reliable model. For instance, information on the biological significance of the E7 expression in these tumours is lacking, but preliminary data indicate that the presence of E7 can improve cell growth rate. Nevertheless, our model holds a valid place in monitoring responses to immunotherapy, as it is orthotopic and derives from naturally occurring OSCC.

In conclusion, we strongly believe that our oral HPV-associated mouse tumour represents an innovative, realistic, and new pre-clinical model for HPV-related OSCC. Moreover, the possibility of applying chemo-radiotherapy to this model together with easy surgical removal of tumour masses offers new perspectives to test current available patient therapy in a pre-clinical model. It is well known that the better prognosis of HPV-positive OSCC could allow less aggressive chemo-radiotherapy, and in this regard, our model will be very useful, and we are performing several protocols of combined therapy (chemo/radio and immunotherapy). Furthermore, the localization of this tumour permits easy access to intratumoural therapy. Preliminary interesting results of E7GGG/SAP DNA intratumoural injection are shown in Fig. 2c, indicating the validity of this model in exploring new promising ways of treatment.

## Concluding remarks

Our studies are aimed at the development of low-cost technologies, such as DNA vaccines as well as plant-produced vaccines, and have proven that these immunotherapies are ready for clinical use. The oral pre-clinical model of HPV-associated cancer will have a pivotal role in designing new combined intervention in HPV-positive OSCC. Ongoing works is aimed at the development of new DNA/plant-derived vaccines against other HPV targets like the E5 oncogene of HR-HPV [57] in association with new emerging biological therapies [58].

## Abbreviations

16E7SH: Shuffled HPV-16 E7
APC: Antigen-Presenting Cells
CIN: Cervical intraepithelial neoplasia
CP: Capsid protein
CTL: Cytotoxic T-lymphocytes
DTH: Delayed-type hypersensitivity
EP: Electroporation
HNC: Head and neck cancer
HPV: Human papillomavirus
HR-HPV: High-risk HPV
Hsp70: Heat shock protein 70
IgG: Immunoglobulins
Ii: Invariant chain
LicKM: Circularly permutated lichenase
MHC: Major histocompatibility complex
N9: Nonoxynol-9
NK: Natural killer
OSCC: Oral squamous cell carcinoma
PVX: Potato virus X
RIPs: Ribosome-inactivating proteins
SAP: Saporin
TAA: Tumour-associated antigens
TAP: Transporter associated with antigen processing
TLR: Toll-like receptors
Treg: Regulatory T-lymphocyte
VLP: Virus-like particles
